# Toward Closing the Loop in Image-to-Image Conversion in Radiotherapy: A Quality Control Tool to Predict Synthetic Computed Tomography Hounsfield Unit Accuracy

**DOI:** 10.3390/jimaging10120316

**Published:** 2024-12-10

**Authors:** Paolo Zaffino, Ciro Benito Raggio, Adrian Thummerer, Gabriel Guterres Marmitt, Johannes Albertus Langendijk, Anna Procopio, Carlo Cosentino, Joao Seco, Antje Christin Knopf, Stefan Both, Maria Francesca Spadea

**Affiliations:** 1 Department of Experimental and Clinical Medicine, Magna Graecia University, viale Europa, 88100 Catanzaro, Italy; anna.procopio@unicz.it (A.P.); carlo.cosentino@unicz.it (C.C.); 2 Department of Radiation Oncology, University Medical Center Groningen, University of Groningen, 9712 CP Groningen, The Netherlands; g.guterres.marmitt@umcg.nl (G.G.M.); j.a.langendijk@umcg.nl (J.A.L.); s.both@umcg.nl (S.B.); 3 Institute of Biomedical Engineering, Karlsruhe Institute of Technology (KIT), 76131 Karlsruhe, Germany; ciro.raggio@kit.edu (C.B.R.); mf.spadea@kit.edu (M.F.S.); 4 Department of Radiation Oncology, University Hospital, LMU Munich, 81377 Munich, Germany; adrian.thummerer@med.uni-muenchen.de; 5 Department of Biomedical Physics in Radiation Oncology, Deutsches Krebsfoschungszentrum (DKFZ), 69120 Heidelberg, Germany; j.seco@dkfz-heidelberg.de; 6 Department of Physics and Astronomy, Heidelberg University, 69120 Heidelberg, Germany; 7 Institute for Medical Engineering and Medical Informatics, School of Life Science FHNW, 4132 Muttenz, Switzerland; antje.knopf@fhnw.ch; 8 Department of Radiotherapy and Radiation Oncology, Faculty of Medicine, University Hospital Carl Gustav Carus, Technische Universität Dresden, 01307 Dresden, Germany

**Keywords:** synthetic CT, conversion prediction, MR-only adaptive radiotherapy, deep learning

## Abstract

In recent years, synthetic Computed Tomography (CT) images generated from Magnetic Resonance (MR) or Cone Beam Computed Tomography (CBCT) acquisitions have been shown to be comparable to real CT images in terms of dose computation for radiotherapy simulation. However, until now, there has been no independent strategy to assess the quality of each synthetic image in the absence of ground truth. In this work, we propose a Deep Learning (DL)-based framework to predict the accuracy of synthetic CT in terms of Mean Absolute Error (MAE) without the need for a ground truth (GT). The proposed algorithm generates a volumetric map as an output, informing clinicians of the predicted MAE slice-by-slice. A cascading multi-model architecture was used to deal with the complexity of the MAE prediction task. The workflow was trained and tested on two cohorts of head and neck cancer patients with different imaging modalities: 27 MR scans and 33 CBCT. The algorithm evaluation revealed an accurate HU prediction (a median absolute prediction deviation equal to 4 HU for CBCT-based synthetic CTs and 6 HU for MR-based synthetic CTs), with discrepancies that do not affect the clinical decisions made on the basis of the proposed estimation. The workflow exhibited no systematic error in MAE prediction. This work represents a proof of concept about the feasibility of synthetic CT evaluation in daily clinical practice, and it paves the way for future patient-specific quality assessment strategies.

## 1. Introduction

Recently, several methods able to convert Magnetic Resonance (MR) and Cone Beam Computed Tomography (CBCT) images into synthetic CT (sCT) have been proposed [1,2,3,4]. This image-to-image (I2I) translation strategy can help when it is not possible or not convenient to acquire a real CT. In particular, radiotherapy is one of the main fields where sCT can play a fundamental role in improving treatment planning, allowing MR and CBCT to be integrated into the imaging pipeline without the need for additional CT scans [5,6,7,8,9,10,11]. Indeed, MR offers better soft tissue contrast to contour the target tissue and the organs at risk during treatment simulation. However, MR intensities do not reveal electron density properties, and additional CT acquisitions are necessary for dose calculation purposes. CBCT, on the other hand, is widely used for assessing inter-fraction geometric deviations in adaptive radiotherapy. But, again, CBCT cannot be used to calculate the dose due to the poor image quality; this forces the clinician to re-plan the treatment on new CT scans when it is necessary. The conversion of MR and CBCT in sCT images can reduce radiation toxicity among patients and decrease the time required for the treatment [2,12]. To date, the best-performing strategies to remap image intensities rely on supervised artificial intelligence algorithms [3,4], in particular those based on deep learning (DL). However, the accuracy of this kind of algorithm strongly depends on the dataset used for the training, and they can underperform in case of strong image artifacts and atypical anatomy. Moreover, the translation quality is assessed by comparing the sCT with the real CT, which represents the ground truth (GT). One of the most used metrics to assess conversion accuracy is the pixel-to-pixel Mean Absolute Error (MAE) [3], defined as Equation (1).

(1) $$MAE=\frac{\sum_{i=1}^{n}\left|GT_{i}-sCT_{i}\right|}{n}$$

$$\begin{array}{c} \mathrm{Mean}\;\mathrm{Absolute}\;\mathrm{Error}\;\mathrm{formula}.\\ i\;\mathrm{ranges}\;\mathrm{from}\;1\;\mathrm{to}\;n,\;\mathrm{number}\;\mathrm{of}\;\mathrm{pixels}/\mathrm{voxels}\;\mathrm{both}\;\mathrm{for}\;\mathrm{GT}\;\mathrm{and}\;\mathrm{sCT}. \end{array}$$

In the case of clinical use, the absence of GT makes it impossible to evaluate the conversion accuracy, which has a direct impact on dose calculation. As a consequence, clinicians have to trust the pre-trained DL algorithms, being unable, except for eye-catching macroscopic mistakes, to detect conversion errors that could dramatically affect the treatment. For the clinical use of sCT for treatment adaptation [13], the implementation of online quality control procedures is mandatory. Some recent works [14,15,16] propose a DL model for synthetic CT generation with uncertainty predictions. The conversion pipeline provides an sCT alongside a predicted uncertainty map. A shortcoming of this approach is the information generation within one algorithm. In case of conversion failure, the uncertainty estimation could also be unreliable. Moreover, it is worth underlining the fundamental difference between “uncertainty” and “error”: a system (as well as a human) can make totally wrong predictions while being, at the same time, absolutely certain about them. For this reason, it is important to predict, preferably by using an independent tool, the conversion error of a process rather than its confidence. In this paper, we implemented a strategy to estimate the quality of sCT in terms of MAE without the need for the corresponding GT and independent of the translation model. The method is based on deep learning algorithms that were trained and tested on two patient cohorts including MR- and CBCT-based sCT images.

## 2. Materials and Methods

### 2.1. Dataset

Two datasets of head and neck cancer patients, previously described in Thummerer et al. [17,18], were used in this study. All patients were treated with intensity-modulated proton therapy at the University Medical Center Groningen, The Netherlands. The first set included 33 patients scanned both with CBCT and CT. The second set included 27 patients with MR and corresponding CT images. All CBCT/CT and MR/CT pairs were carefully aligned using deformable image registration to match the anatomy. For each patient of both datasets, an sCT was generated by using a basic version of the algorithm described in Spadea et al. [19]. The original Spadea et al. method, relying on an encoder/decoder architecture to execute 2D-based image translation, takes advantage of a multi-plane approach, in which volumes obtained along axial, sagittal, and coronal directions are voted to generate the final image. In this work, only the synthetic CTs reconstructed by stacking the translated 2D images along the craniocaudal axis were used. From now on, the sCTs derived from CBCT will be referred to as $sCT_{CBCT}$ and the ones obtained from MR as $sCT_{MR}$.

### 2.2. General Pipeline

**Figure 1:**
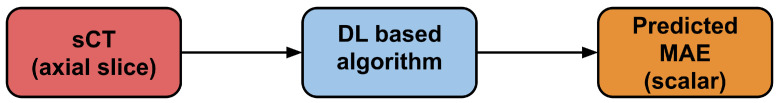
Representation of the general MAE prediction pipeline. An axial sCT slice is given as input, and the associated MAE scalar for the image slice is predicted by using a DL pipeline.

The proposed quality control approach is based on a 2D DL model architecture pipeline. The main idea is to provide an axial sCT slice as the input to the DL scheme and to obtain the predicted MAE for that image as the output (see Figure 1). To achieve this, the DL model is trained on retrospective data where ground truth CT and the corresponding sCT are available. Having GT images, the actual MAE can be computed slice-by-slice according to equation 1. During the training, the pipeline receives the sCT slice (input) and the actual MAE (output to predict). Afterwards, during deployment, the trained models only receive the sCT slices and predict an MAE scalar for the synthetic 2D image.

**Figure 2:**
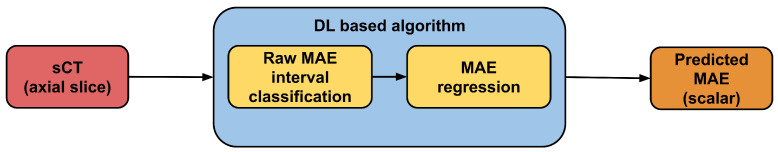
A more detailed graphical representation of the MAE prediction pipeline. The final MAE prediction is obtained as a result of two DL steps: First a raw MAE interval classification is performed, followed by a more precise MAE estimation based on a regression algorithm.

More specifically, as depicted in Figure 2, the workflow consists of a cascade of 2 series of DL models, used in a sequential way to handle the complexity of the MAE prediction task. The rationale is to first execute a raw prediction of the MAE interval (classification), followed by a more precise estimation of the MAE value (regression). This approach was inspired by a similar and successful strategy to predict segmentation accuracy in multi-atlas-based segmentation [20]. This strategy allows the initial problem to be split into two subproblems, both of which are easier to solve than the first one.

### 2.3. Output Visualization

**Figure 3:**
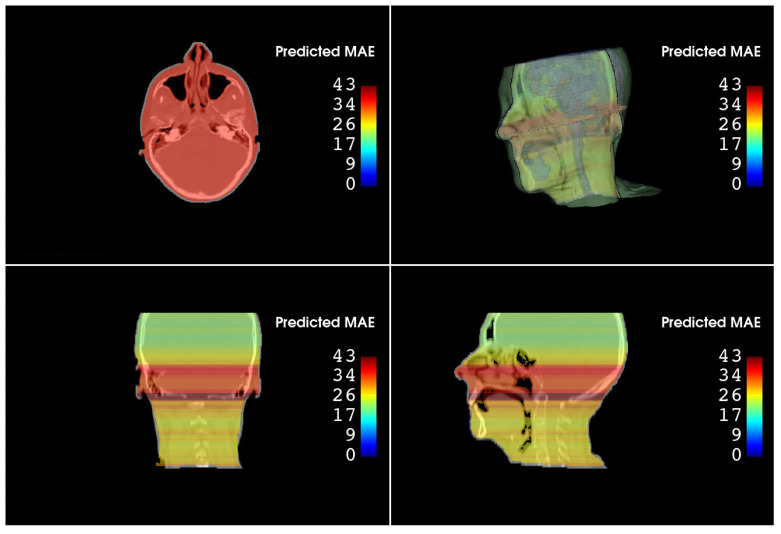
Exemplary $sCT_{CBCT}$ overlaid with its $pMAE_{volume}$. In addition to the 2D views (axial, sagittal, and coronal planes), the 3D representation is also shown.

The output of a single prediction is a scalar value representing the estimated MAE for the entire given axial slice. In envisioning a clinical use, it is fundamental to visualize the predicted conversion error slice-by-slice and overlaid on the sCT. To achieve this, a volume (named “Predicted MAE volume”, $pMAE_{volume}$), having the same shape as the corresponding sCT, is generated. During the sCT evaluation step, the predicted MAE of the i-th axial slice is assigned to the i-th axial slice of $pMAE_{volume}$, which will be visualized by taking advantage of a dedicated color map. Figure 3 shows an example of an sCT overlaid to its $pMAE_{volume}$.

### 2.4. Pipeline Details

**Figure 4:**
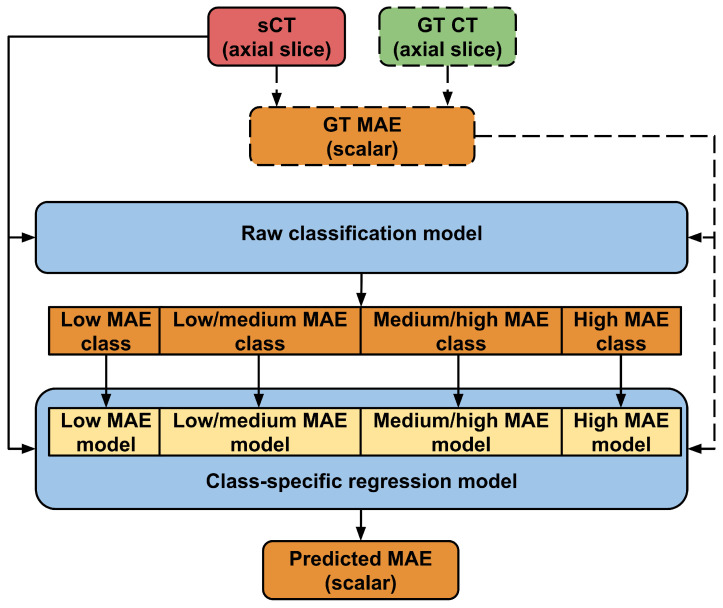
Detailed workflow of MAE prediction. A single sCT axial slice is fed firstly into a DL model that classifies it as belonging to a specific MAE class. According to this prediction, the 2D image is then provided as input to a connected DL regression model, specifically trained to operate on a restricted range of MAE values. As a result, the MAE of a single sCT slice can be forecasted. In order to train the different models with a GT MAE, the ground truth CT is needed (dashed lines are needed only to train the models).

As already reported, the first step for MAE estimation is a raw classification of the conversion error. More specifically, 4 classes are used in the proposed pipeline (low, low-medium, medium-high, and high MAE). In order to define the MAE range for each interval, the actual MAE distribution of the entire dataset is computed. Then, the first class of the prediction is associated with an MAE ranging from 0 to the 1st quartile of the total distribution, the second class from the 1st to the 2nd quartile, the third class from the 2nd to the 3rd quartile, and finally the fourth class from the 3rd percentile to the highest MAE values (in order to deal with an open interval, this last bin was saturated to an upper bound HU value). This allows for the balancing of the number of examples per class. The same values are also used as an initialization to define the MAE boundaries for the regression step (the second one of the pipeline), where a separate model is trained for each of the four MAE classes. More specifically, each of the four regression models is trained by showing only the slices having an MAE value included in the same range of the connected classification class. However, in order to take into account possible mistakes in the classification step, the boundaries of the regression models are relaxed by also including cases with GT MAE slightly lower/higher than the quartiles (±5 HU). Table 1 reports the computed bin ranges, both for the classification and regression steps. In total, 5 models have to be trained (1 for classification and 4 for regression), but, in the inference mode, each sCT slice will be processed just by 2 of them (classification followed by the class-specific regression). For the sake of clarity, the entire prediction pipeline is shown in Figure 4.

### 2.5. DL Architecture and Training

The DL architecture to be used in this work was chosen after testing several models, both from VGG [21] and ResNet [22] families. Our tests revealed that VGG-16 provides the best prediction accuracies, so it was used as the baseline architecture for all the models. VGG (Visual Geometry Group) is a family of high-performance convolutional neural networks developed for classification and regression tasks. Even though it was created for general-purpose computer vision applications, it has also been widely used in the biomedical environment. The backbone of VGG-16 includes 13 convolutional layers (that serve as feature extraction engines), followed by 3 fully connected layers (that are used to obtain the output label starting from the computed feature map). The loss functions to optimize for classification and regression are, respectively, cross entropy and mean squared error. The best epoch for the classification task is not chosen on the basis of the cross entropy value but by considering the accuracy weighted according to the misclassification magnitude. In practical terms, this means that a classification error of 1 class is preferred over 2 and 3 classes of misclassification. This choice is made to let the regression step, trained on slightly larger MAE boundaries than the connected classification bin, recover the potential classification error. The entire workflow is written in Python by taking advantage of PyTorch library [23]. The learning rate is set to $5\times 10^{-5}$, and L1 and L2 regularizations are included (both weights equal to $4\times 10^{-4}$). The image augmentation scheme includes mirroring and translations, as defined by Spadea et al [19]. The dataset is split into 75% training, 10% validation, and 15% testing. K-fold cross-validation is used to evaluate the model generalization performance on the entire dataset.

### 2.6. Experiments

To assess the learning generalization capability of the approach toward different image modalities, 3 pipelines are implemented:

1. Models trained only with $sCT_{CBCT}$ as input (named $Pipeline_{CBCT}$),
2. Models trained only with $sCT_{MR}$ as input ($Pipeline_{MR}$),
3. Models trained with both $sCT_{CBCT}$ and $sCT_{MR}$ as input ($Pipeline_{MIXED}$).

Once all the models of each pipeline are trained, multiple testing schemes are run to evaluate the prediction performance. In particular, the following experiments are executed:

- $Pipeline_{CBCT}$ is used to predict the MAE only for $sCT_{CBCT}$ data
- $Pipeline_{MR}$ is used to predict the MAE only for $sCT_{MR}$ data
- $Pipeline_{MIXED}$ is used to predict the MAE only for $sCT_{CBCT}$ data
- $Pipeline_{MIXED}$ is used to predict the MAE only for $sCT_{MR}$ data

Table 1 shows the MAE binning used to train both classification and regression models in each pipeline.

### 2.7. Prediction Pipeline Evaluation

The accuracy of the prediction workflow is quantified in terms of signed and unsigned deviation from the GT MAE. More specifically, both the Prediction Deviation (PD) and the Absolute Prediction Deviation (APD) are computed for each slice as defined in Equations (2) and (3):

(2) $$PD=MAE_{GT}-MAE_{predicted}$$

SignedpredicteddeviationbetweenGTandinferredMAE.

(3) $$APD=\left|MAE_{GT}-MAE_{predicted}\right|$$

AbsolutepredicteddeviationbetweenGTandinferredMAE.

Both $MAE_{GT}$ and $MAE_{predicted}$ are saturated to the upper bound values reported in Table 1.

## 3. Results

**Figure 5:**
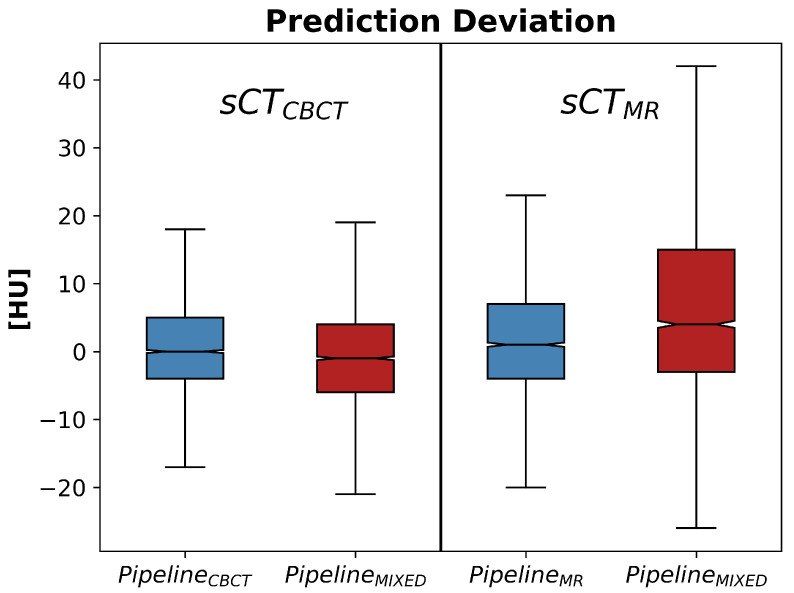
PD distributions for modality-specific and mixed pipelines. Results for $sCT_{CBCT}$ and $sCT_{MR}$ are reported, respectively, in the left and in the right panel.

**Figure 6:**
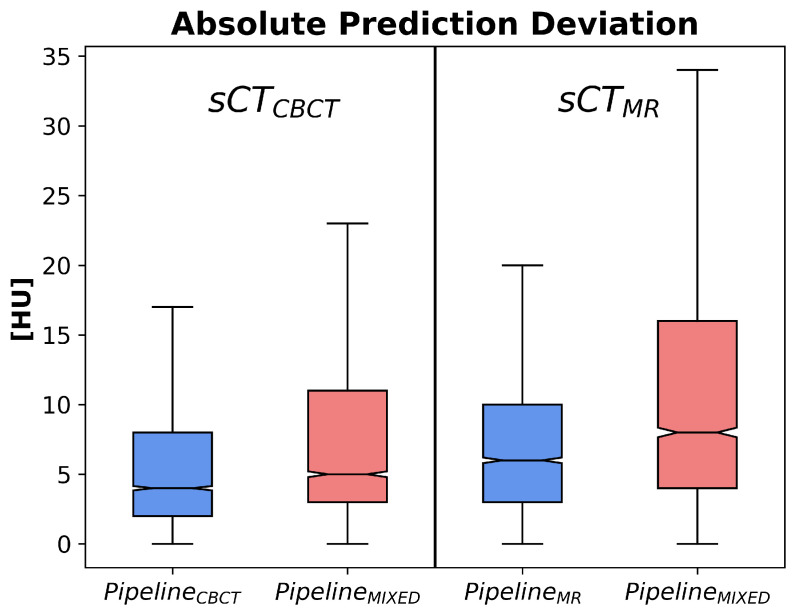
APD distributions for modality-specific and mixed pipelines. Results for $sCT_{CBCT}$ and $sCT_{MR}$ are reported, respectively, in the left and in the right panel.

Figure 5 and Figure 6 show, for each DL pipeline, the PD and APD distributions computed over all the 2D slices of all the patients.

Tables containing the median, the 5th, the 25th, the 75th, and the 95th percentiles of both PD and APD are reported in Appendix A. To assess whether a statistical difference exists between the predictions obtained by using the mixed-trained workflow ($Pipeline_{MIXED}$) versus the specific modality pipeline ($Pipeline_{CBCT}$ and $Pipeline_{MR}$), the Wilcoxon rank-sum test is run over all the pairs of MAE predictions obtained for all the patients fed into the different pipelines. These tests reveal that both single-modality options performed significantly better than the mixed solution (*p*-values < 0.01). Figure 3 shows an example of the overlay of the sCT and its $pMAE_{volume}$. As can be seen, for slices depicting small and movable anatomical structures (e.g., nasal cavities), higher MAE values are predicted. This behavior was expected and desirable, confirming the effectiveness of the proposed strategy.

Despite the necessity for a more extended training phase to enable the training of 5 different models, the inference step is completed in a few seconds. As a result, the required time to evaluate an sCT is totally compatible with a clinical scenario and will not slow down the radiotherapy planning workflow.

## 4. Discussion

In this work, we introduce a new method to predict the accuracy of sCT generation when the GT is not available (i.e. use of sCT in a clinical setting). The technique is based on using a DL model that is independent from any other model used to generate the sCT.

Being the first implemented workflow for the autonomous MAE prediction of DL-based sCT, there are no closely related studies to compare it with. The most similar articles in the literature are those that generate uncertainty maps alongside sCT [14,15,16]. The idea behind such works is to have a distribution of values for each voxel rather than a single intensity (by using Bayesian neural networks or by executing multiple inferences with active dropout layers). The averages of the obtained distributions represent the final sCT, while the variabilities are informative about the uncertainty of the prediction (with the uncertainty map correlating with the GT intensity and dose errors). However, in this regard, it is fundamental to underline the conceptual difference existing between our proposed workflow (conversion error estimation) and the uncertainty prediction proposed by others, since being confident about something does not necessarily mean being accurate. The main concern about these strategies is the possibility that the conversion model provides, with high assurance, a synthetic image that is actually wrong. Because of this, the main pillars of the proposed workflow are the separation between the image translation step and its quality control, and the prediction of the actual error rather than the confidence. Predicting the conversion error of sCT, in fact, is of paramount importance in contexts such as radiotherapy, where wrong HU assignments lead to wrong dose estimation to the target and organs at risk. Specifically, for the clinical introduction of MR-guided radio and proton therapy [24], an autonomous and independent quality control tool (an information with an additional level of redundancy and with a rationale totally different from the uncertainty assessment produced by the conversion algorithm itself) would be required to ensure reliable clinical decision-making based on deep-learning generated medical image data. We would like to highlight that the aim of the proposed tool is to intercept conversion errors that are not expected and not detectable by the human operator. Clinicians, in fact, well know the anatomical regions that can be more likely affected by translation error and are able to identify evident anomalous conversions, so our intention is to catch unforeseeable failures. The findings of our experiments show that it is possible to predict the accuracy of sCT in terms of MAE, with small deviations between inferred and GT MAE values. In fact, considering the HU range, an absolute prediction deviation of 4±3HU (for CBCT-derived sCT) and 6±3.5HU (for MR-derived sCT) has no impact on the dose estimation, even in the case of proton therapy, known to be extremely sensitive to HU variations. Signed prediction deviations demonstrate the absence of systematic errors. Results are also consistent with real-case expectations. Referring to Figure 3, worse conversion error is predicted for axial slices included between the nasal cavities and the mandibular bone. This result is very common in sCT generation, since the image quality in this anatomical area is deteriorated by the presence of dental fillings and motion artifacts. In a typical clinical setting, the user would receive a warning on which part of the sCT is reliable and which is not. In light of the magnitude of the predicted error and its spatial localization with respect to the tumor and the tissues to be spared, clinicians may elect to exclude the use of the sCT. The comparison between models trained by using single-modality versus mixed-modality dataset reveals that better results are obtained with single-modality training. The mixed setup was implemented to test the capability of the workflow to be agnostic to the initial image modality. The entire workflow is based on the VGG-16 model, which has been demonstrated to be the most effective architecture for predicting the MAE in our tests. In addition to its high predictive accuracy, this model requires fewer computational resources than deeper networks, allowing for fast sCT evaluation even in the absence of dedicated high-end GPUs, which is a crucial advantage in a real clinical setting.

Regarding the potential error propagation between the classification and regression steps, the proposed workflow is also robust to this possibility. In fact, as can be deduced from the results (Figure 5 and Figure 6), the prediction deviations are much smaller than the differences that would be generated if the raw MAE bin was misclassified and if this error was not recovered in the regression phase.

The proposed approach could be further improved by replacing the global axial MAE scalars currently predicted as quality control metrics with a more informative index from a clinical point of view. Such an index could, for example, include dosimetric and/or voxel-level prediction. In the future, it would be interesting to evaluate the proposed workflow on images collected in different institutions and converted by using different translation strategies (including cycle-GAN architectures, to get rid of the error introduced by the image registration step). We envision pursuing work in that direction, executing more complex and more computationally demanding experiments. The proposed idea, in addition to enabling a real-time synthetic image evaluation in the clinic, can be also used to implement synthetic image generation algorithms, when CT-MRI or CT-CBCT paired data are not available. The pipeline described here, in fact, can directly replace the computation of the MAE-based loss when the ground truth CT is not available.

## 5. Conclusions

In conclusion, we demonstrated that an independent prediction of the performance of an algorithm for sCT generation is possible and, most importantly, we hope to start a debate about usable strategies in real clinical environments for assessing the quality of synthetic medical images.

## Figures and Tables

**Table 1:** MAE ranges for classification and regression, both for MR, CBCT, and MIXED pipelines.

Pipeline	Low MAE	Medium-Low MAE	Medium-High MAE	High MAE
MR	0–47 (classification)	47–54 (classification)	54–68 (classification)	68–100 (classification)
	0–52 (regression)	42–59 (regression)	49–73 (regression)	63–100 (regression)
CBCT	0–27 (classification)	27–32 (classification)	32–42 (classification)	42–70 (classification)
	0–32 (regression)	22–37 (regression)	27–47 (regression)	37–70 (regression)
MIXED	0–32 (classification)	32–44 (classification)	44–56 (classification)	56–90 (classification)
	0–37 (regression)	27–49 (regression)	39–61 (regression)	51–90 (regression)

## Data Availability

The datasets used in this article are not readily available because they contain sensitive information. Requests to access the datasets should be directed to the corresponding author.

## Appendix A

Table A1 and Table A2 show, for each DL pipeline, the median and the percentiles of PD and APD distributions computed over all the 2D slices of all the patients.

**Table A1:** Statistics about the PD for each conducted experiment.

	Prediction Deviation (PD)				
Experiment	5th Percentile [HU]	25th Percentile [HU]	Median [HU]	75th Percentile [HU]	95th Percentile [HU]
$Pipeline_{CBCT}$ predicting on $sCT_{CBCT}$	−10	−4	0	5	20
$Pipeline_{MR}$ predicting on $sCT_{MR}$	−12	−4	1	7	16
$Pipeline_{MIXED}$ predicting on $sCT_{CBCT}$	−23	−6	−1	4	21
$Pipeline_{MIXED}$ predicting on $sCT_{MR}$	−11	−3	4	15	32

**Table A2:** Statistics about the APD for each conducted experiment.

	Absolute Prediction Deviation (APD)				
Experiment	5th Percentile [HU]	25th Percentile [HU]	Median [HU]	75th Percentile [HU]	95th Percentile [HU]
$Pipeline_{CBCT}$ predicting on $sCT_{CBCT}$	0	2	4	8	21
$Pipeline_{MR}$ predicting on $sCT_{MR}$	1	3	6	10	17
$Pipeline_{MIXED}$ predicting on $sCT_{CBCT}$	0	3	5	11	29
$Pipeline_{MIXED}$ predicting on $sCT_{MR}$	1	4	8	16	32
